# Atomic force microscopy reveals new biophysical markers for monitoring subcellular changes in oxidative injury: Neuroprotective effects of quercetin at the nanoscale

**DOI:** 10.1371/journal.pone.0200119

**Published:** 2018-10-10

**Authors:** Maja Jazvinšćak Jembrek, Josipa Vlainić, Vida Čadež, Suzana Šegota

**Affiliations:** 1 Division of Molecular Medicine, Ruđer Bošković Institute, Zagreb, Croatia; 2 Department of Psychology, Croatian Catholic University, Zagreb, Croatia; 3 Division of Physical Chemistry, Ruđer Bošković Institute, Zagreb, Croatia; LAAS-CNRS, FRANCE

## Abstract

Oxidative stress has been recognised as an important pathological mechanism underlying the development of neurodegenerative diseases. The biomarkers for assessing the degree of oxidative stress have been attracting much interest because of their potential clinical relevance in understanding the cellular effects of free radicals and evaluation of the efficacy of drug treatment. Here, an interdisciplinary approach using atomic force microscopy (AFM) and cellular and biological molecular methods were used to investigate oxidative damage in P19 neurons and to reveal the underlying mechanism of protective action of quercetin. Biological methods demonstrated the oxidative damage of P19 neurons and showed that quercetin improved neuronal survival by preventing H_2_O_2_-induced p53 and Bcl-2 down-regulation and modulated Akt and ERK1/2 signalling pathways. For the first time, AFM was employed to evaluate morphologically (roughness, height, Feret dimension) and nanomechanical (elasticity) properties in H_2_O_2_-induced neuronal damage. The AFM analysis revealed that quercetin suppressed H_2_O_2_-provoked changes in cell membrane elasticity and morphological properties, thus confirming its neuroprotective activity. The obtained results indicate the potential of AFM-measured parameters as a biophysical markers of oxidative stress-induced neurodegeneration. In general, our study suggests that AFM can be used as a highly valuable tool in other biomedical applications aimed at screening and monitoring of drug-induced effects at cellular level.

## Introduction

Oxidative stress represents a common mechanism of neuronal death in a variety of neuropathologies, including neurodegenerative diseases such as Alzheimer's disease and Parkinson disease [1]. The brain is particularly vulnerable to oxidative stress due to its large oxygen consumption, high content of polyunsaturated fatty acids, accumulation of redox-reactive transient metal ions and limited endogenous antioxidant protection [2].

At physiological levels ROS act as signalling molecules, but when present in excess they may induce an oxidative stress response and trigger cell death by modulating redox-sensitive signalling pathways and gene expression. Among different ROS molecules, H_2_O_2_ is considered as the key target in neuroprotection as one of the most abundant ROS in aerobic organisms. Moreover, it can be converted to more toxic species of which hydroxyl radical is particularly dangerous [3]. The mechanism of the H_2_O_2_-mediated signaling relies on the oxidation of redox-sensitive thiol groups in cysteine residues of different target enzymes and transcription factors, thereby modulating their functions [4]. At concentrations above the physiological threshold, H_2_O_2_ can change activities of different signalling cascades, such as pathways of mitogen-activated protein kinases (MAPK) and protein kinase B (PKB/Akt). This ultimately triggers specific nuclear or cytoplasmic response, often ending in cell death [5,6]. In addition to modulation of intracellular transduction pathways, increased levels of ROS may induce damage to all biological macromolecules that further exacerbates neuronal death [2].

Quercetin, a plant-derived polyphenolic nutraceutical, possesses a wide spectrum of health-promoting effects mainly attributed to its strong antioxidative capacity. It is effective against various oxidants and other neurotoxic molecules that induce oxidative stress and mimic the pathological hallmarks of neurodegenerative diseases [7,8]. *In vitro*, quercetin exerts its neuroprotective effects acting as a potent direct radical scavenger and a metal chelator but is also able to downregulate redox-sensitive signalling [9,10]. However, due to low bioavailability, the expected concentrations of quercetin in the brain tissue are below those that are required for direct antioxidant activity. Furthermore, concentrations of endogenous antioxidants greatly surpass anticipated levels of quercetin in the brain. Hence, it is suggested that the neuroprotective effects of quercetin *in vivo* are not achieved through direct ROS scavenging [8]. Instead, it seems that the modulation of intracellular signalling pathways probably represents a primary mode of quercetin action *in vivo* [11,12]. Assumption to hypothesis, it has been shown that structural characteristics of quercetin molecule involved in neuroprotective action differ from those that provide free radical scavenging [13].

Quercetin accumulates in the nucleus, at distinct loci, where may affect transcription and/or activity of numerous transcription factors [14,15]. Quercetin also may act inhibitory on a number of kinases signalling pathways including Akt/PKB, extracellular signal-regulated protein kinases (ERK) 1/2 and c-Jun N-terminal kinase (JNK) [11,16,17]. The stimulatory effects on the same kinases WERE also demonstrated, leading to the expression of survival and defensive genes [10,18]. Hence, the exact mechanism of neuroprotective effects of quercetin remains puzzling, particularly when considering modulation of intracellular signalling pathways.

Physiological functions of a cell are closely related to its morphological characteristics [19,20]. When pathological toxin- and drug-induced molecular changes occur inside the cell, its overall morphology usually changes as well. Recent studies have shown that the nanomechanical behaviour of the cell plays an important role in maintaining cellular physiological functions and also presents a novel biomarker for indicating different cell states [21]. In the present study, we implemented a novel experimental approach, combining molecular biology with atomic force microscopy (AFM) as an advanced tool, to obtain information about drug-induced neuronal changes during oxidative stress. The AFM applications in neuronal research is developing and it is primarily used for quantitative imaging of cell surface topography. In non-imaging mode, AFM provides spatially resolved maps of the nanomechanical characteristics usually reported as cell elasticity [22,23]. Beside studying individual cells, AFM was employed for nanomechanical phenotyping of the murine brain during maturation [24]. Both imaging and non-imaging AFM modes can bring specific information about neuronal membranes and cytoskeleton architecture. Structural and nanomechanical properties of neurons are highly affected by environmental conditions that can change the organization of their microtubules, actin filaments and neurofilaments [23,25]. AFM was already utilized for detecting nanoscopic changes resulting from oxidative damage in the plasma membrane of glioblastoma cells [26].

The aim of the present study was to combine capabilities of AFM with molecular biology tools to better understand cellular and molecular consequences of H_2_O_2_-induced oxidative injury in P19 neurons and to investigate molecular mechanisms of quercetin-mediated neuroprotection that falls beyond its antioxidant activity. For the first time, we used AFM to screen subtle changes of neuronal membrane topography and nanomechanical properties induced by drug treatment during the oxidative insult.

## Materials and methods

### Chemicals and reagents

*All-trans* retinoic acid (ATRA), cytosine-arabinofuranoside, poly-L-lysine, 1,4-diamino-2,3-dicyano-1,4-bis[2-aminophenylthio] butadiene (UO126) and all chemicals used for maintaining and differentiation of P19 cells (unless otherwise stated) were purchased from Sigma-Aldrich Chemicals (St. Louis, MO, USA). Quercetin dihydrate was obtained from Aldrich Ch. Co. Inc. (Milwaukee WI, USA), wortmannin was purchased from Ascent Scientific (Princeton, NJ, USA) and hydrogen peroxide solutions were obtained from Kemika (Zagreb, Croatia) and Sigma-Aldrich Chemicals (Cat. No. 216763, stabilized solution containing inhibitors). All other chemicals used were of analytical grade.

### P19 cell culturing and P19 neuronal differentiation

Undifferentiated P19 cells (pluripotent mouse teratocarcinoma cell line) were cultured in high-glucose Dulbecco's modified Eagle's medium (DMEM) containing 10% heat-inactivated fetal bovine serum (FBS), 2 mM L-glutamine, 100 units/ml penicillin G and 100 μg/ml streptomycin (growth medium) in a humidified atmosphere of 5% CO_2_ at 37°C. They were passaged every two days using trypsin (0.05% trypsin, 1 mM EDTA) in phosphate buffered saline (PBS).

For induction of neuronal differentiation, exponentially growing P19 cells (1x10^6^) were seeded into non-adhesive bacteriological-grade Petri dishes (10 cm) containing 10 ml of DMEM medium supplemented with 5% FBS, 2 mM L-glutamine, antibiotics and 1μM ATRA (induction medium). Embryonal bodies of P19 cells were formed in 1–2 days. After 48 h, the old medium was replaced with the fresh ATRA-containing medium and aggregated cultures were grown for two more days. After the four-day of ATRA treatment, P19 embryonal bodies were harvested, washed with PBS, trypsinized, collected by centrifugation (200 g, 5 minutes), and resuspended in growth medium. For optimal neuronal differentiation single cells at a density of 10^5^ cells/cm^2^ were plated onto 96-well plates or 35 mm Petri culture dishes (Cell^+^, Sarstedt, Newton, NC, USA and NUNC, Roskilde, Denmark), and grown in growth medium for two more days. Finally, the growth medium was replaced with serum-free medium containing DMEM supplemented with insulin, transferrin, selenium and ethanolamine solution (ITS-X, Gibco), 2 mM L-glutamine and antibiotics (neuron-specific medium), and cells were grown for additional 2 days in the presence of 10 μM mitotic inhibitor cytosine-arabinofuranoside (AraC) to inhibit proliferation of non-neuronal cells. Complete neuronal maturation was confirmed with monoclonal anti-tubulin β-III mouse IgG, clone TU-20 conjugated with Alexa Fluor 488 (Millipore, Temecula, CA) as previously described [27]. Differentiated cells expressing neuronal marker β-III tubulin were visualized by fluorescence microscopy. Completely differentiated P19 neurons exert morphological, neurochemical and electrophysiological properties resembling neurons from the mammalian brain, and represent an established model for pharmacological studies [28,29].

### Drug treatment

In all experiments, P19 neurons were treated 8 days after the initiation of the differentiation procedure (DIV8). Each batch of cultured cells was divided into control and drug-treated groups. For inducing oxidative damage, P19 neurons were incubated with 1.5 mM H_2_O_2_ in the neuron-specific medium for 24 hours, alone or in the presence of various concentrations of quercetin that failed to affect the viability of P19 neurons when applied alone [29]. To examine the effects of quercetin on kinase signalling pathways, P19 neurons were pretreated with UO126 or wortmannin for 60 min and then exposed to H_2_O_2_, inhibitor and 150 μM quercetin for additional 24 hours.

### Assessment of cell death

#### Trypan blue exclusion assay

The viability of P19 neurons in the presence of 1.5 mM H2O2 was analysed by trypan blue exclusion assay. The method is based on the principle that healthy cells effectively exclude the dye from their cytoplasm, while those with damaged membranes lose this ability and appear blue. Following treatment, culture mediums with floating cells were collected in a centrifuge tube. Attached cells were trypsinized for 5 min, resuspended and pooled with the corresponding medium. Samples were centrifuged at 250 g for 5 min; pellets were resuspended in 250 μl of neuron-specific medium and incubated for 5 min in the presence of 0.4% trypan blue solution. The ratio of trypan blue stained nuclei over the total number of cells was used to determine the percentage of cell death. For each examined group at least 500 neurons were counted by two different investigators.

#### MTT assay

Effects of inhibitors of Akt and ERK1/2 signalling, wortmannin and UO126, respectively, on the neuroprotective effect of quercetin, were determined by 3-(4,5-dimethylthiazol-2-yl)-2,5-diphenyl tetrazolium bromide (MTT) assay. MTT assay was also employed to determine the effects of different concentrations of H_2_O_2_ on the viability of P19 neurons. Estimation of viability is based on the ability of P19 neurons to cleave dissolved MTT into an insoluble formazan product by cleavage of the tetrazolium ring by dehydrogenase enzymes. Briefly, P19 neurons were seeded on 96-well micro-plates and were incubated for 24 hours with H_2_O_2_, quercetin and inhibitors. At the end of the treatment schedule, the medium was removed, and the cells were incubated with 40 μl of MTT solution (0.5 mg/ml final) for 3 h at 37°C. Precipitated formazan was dissolved by adding 160 μl of dimethyl sulfoxide (DMSO). Optical densities of coloured solutions in each well were determined by an automatic ELISA reader at 570 nm. The data were analysed after blank subtraction from all absorbance readings and viability of P19 neurons was calculated according to the following equitation: % cytotoxicity = [A_control_-A_treatment_]/A_control_ x 100.

#### Measurement of reduced glutathione

Reduced glutathione (GSH) is one of the major non-enzymatic intracellular antioxidant defence mechanisms. Changes in the intracellular level of GSH were monitored by using a GSH-Glo Glutathione Assay (Promega, Madison, WI, USA) based on the conversion of a luciferin derivative into luciferin by glutathione S-transferase (GST) in the presence of GSH. Hence, the signal generated in a coupled reaction with luciferase is proportional to the amount of GSH present. According to the manufacturer’s instruction, following treatment, the medium was removed and 100 μl of GSH-Glo reagent was added per well. After a 30-minute incubation, 100 μl of luciferin detection reagent was further added and following 15-minute incubation emitted light was measured in the luminometer (Fluoroskan Ascent FL, Thermo Scientific).

#### Determination of Bcl-2, Bax, p53 and GAPDH mRNA levels by semi-quantitative RT-PCR

Expressions of Bcl-2, Bax, p53 and GAPDH mRNA were examined by semiquantitative RT-PCR analysis according to the method previously described by Jazvinšćak Jembrek and co-workers [29]. cDNAs were amplified and analysed during two consecutive cycles in the log phase of PCR reactions. PCR primers, annealing temperatures, and numbers of cycles are shown in Table 1. The reactions were performed in a Perkin Elmer 9600 thermocycler. Amplified products (10 μl) were electrophoretically separated on a 1.5% agarose gel and stained with ethidium bromide (0.5μg/ml) for 20 minutes. Optical densities of detected bands were analysed using ImageJ NIH software 1.0. Expression of housekeeping gene TATA-binding box protein (TBP) mRNA was used as an internal standard for normalization.

**Table 1:** Primer sequences and conditions used for PCR amplifications.

Gene	Primer sequence	Product length (bp)	Annealing temp. (°C)	Number of cycles
	(5’ **→** 3’)			
Bcl-2	F: GGAGATCGTGATGAAGTACATAC	373	58	27–28
	R: CCTGAAGAGTTCCTCCACCACC			
Bax	F: ATCGAGCAGGGAGGATGGCT	470	62	27–28
	R: CTTCCAGATGGTGAGCGAGG			
p53	F: AGAGACCGCCGTACAGAAGA	231	62	31–32
	R: CTGTAGCATGGGCATCCTTT			
TBP	F: ACCCTTCACCAATGACTCCTATG	190	60	29–30
	R: ATGATGACTGCAGCAAATCGC			
GAPDH	F: ACCACAGTCCATGCCATCAC	452	60	24–25
	R: TCCACCACCCTGTTGCTGTA			

### AFM measurements

All cell imaging and force mapping measurements were obtained on the JPK NanoWizard ULTRA Speed and JPK NanoWizard 4 AFM system coupled to the Nikon Eclipse TE2000-U inverted optical microscope. Cantilever qp-Bio-AC with the nominal spring constant of 0.05–0.07 N/m was used for measuring in liquid. The measurements are performed in PBS with the Petri dish fixed to the standard sample holder. All images were acquired from fixed neurons to provide more accurate measurements of the height and structure of the individual subcellular regions using QI mode. Neurons grown on Petri dishes were fixed with 4% paraformaldehyde (15 min RT) that is conventionally used for biological applications [30].

The Petri dishes with the sample cells were sealed on the standard sample holder using two-component rubber glue. Before imaging, neurons were examined with an inverted microscope, fresh PBS was added, and cells of interest were selected. For each group, we prepared 3 different samples and analysed 9 neurons. The focus was on neurons with a round cell body. Furthermore, morphological properties of the distinct soma regions were determined at the optimized imaging conditions to avoid any possible influence of the applied force on imaging. During scanning both trace and retrace images were recorded and compared for accuracy. No substantial difference could be observed between them. For each sample, an overview scan of the whole cell was acquired, as well as a detailed image of 2 μm x 2 μm with 256 x 256-pixel resolution. For all measurements, a setpoint of 400 pN and an extend/retract speed of 110–195 μms^-1^ was used. For the overview image of the control sample, a setpoint of 500 pN was used. Prior to measuring, the cantilever was calibrated using the in-built non-contact method [31] The data were analysed using the JPK Data Processing software.

The roughness was calculated for the raw height image and for the height image treated with a quadratic plane fit in order to minimize the influence of the cell surface curvature on the roughness values. The plane fit calculates a plane of the desired order from the raw image and subtracts this plane from the whole image. Roughness Average, Ra, is the arithmetic average of the absolute values of the profile heights over the evaluation length. It is defined as

$$R_{a}=\frac{1}{n}\sum_{i=1}^{n}\left|y_{i}\right|$$ (1)

where *y*_i_ is vertical deviations height profiles from the mean plane, while RMS Roughness, *R*_q_, is the root mean square average of the profile heights over the evaluation length defined as:

$$R_{q}=\sqrt{\frac{1}{n}\sum_{i=1}^{n}y_{i}^{2}}$$ (2)

The Young’s modulus was determined using the Hertz model for conical indenters.

### Statistical analysis

Statistical analysis of the data was carried out using GraphPad Software (San Diego, CA). All values are represented as mean ± SEM from at least three independent experiments. Comparisons between group means were evaluated by one-way analysis of variance (ANOVA), and when statistically significant, post hoc analysis with Dunnett’s multiple comparison test (for analysing effects of H_2_O_2_ and quercetin alone) or Tukey's test (for analysing neuroprotective effects of quercetin) followed. The P values less than 0.05 were considered statistically significant.

## Results and discussion

### Biological approach reveals neuroprotective effects of quercetin in H_2_O_2_—induced injury

**Fig 1:**
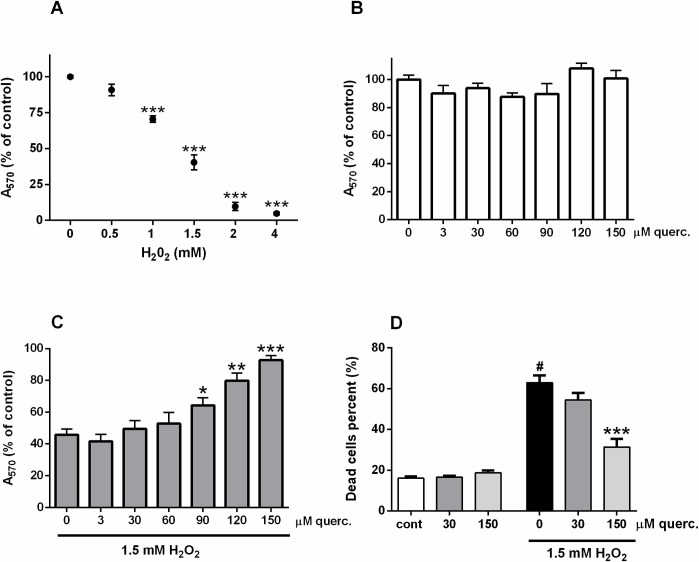
Quercetin improved viability of P19 neurons exposed to H_2_O_2_. In a dose-dependent manner, H_2_O_2_ reduced the viability of P19 neurons (A). Quercetin failed to modify neuronal viability when applied alone up to 150 μM concentration (B). Oxidative injury of P19 neurons was induced by exposure to 1.5 mM H_2_O_2_. Cell death of damaged neurons was analysed in the presence of various concentrations of quercetin by MTT assay (C) or trypan blue exclusion assay (D). Data are expressed as means ± SEM from four to five independent experiments. ^#^P < 0.0001 *vs*. cont; *P < 0.05, **P < 0.001 and ***P < 0.0001 vs. 0 (ONE-way ANOVA followed by Tukey's or Dunnett's multiple comparison test).

In a concentration-dependent manner, exposure to H_2_O_2_ decreased the viability of P19 neurons (Fig 1A; F(5,8) = 169.8, P < 0.0001), whereas they tolerated relatively high concentrations of quercetin without a decrease in survival (Fig 1B). As determined by MTT method (Fig 1C; F(6,28) = 15.80, P < 0.0001) and trypan blue exclusion assay (Fig 1D; F(5,24) = 59.49, P < 0.0001), quercetin applied together with 1.5 mM H_2_O_2_ improved survival of P19 neurons indicating its neuroprotective effects against neuronal death in oxidative conditions.

### Quercetin did not improve H_2_O_2_-induced decrease in glutathione (GSH) content

**Fig 2:**
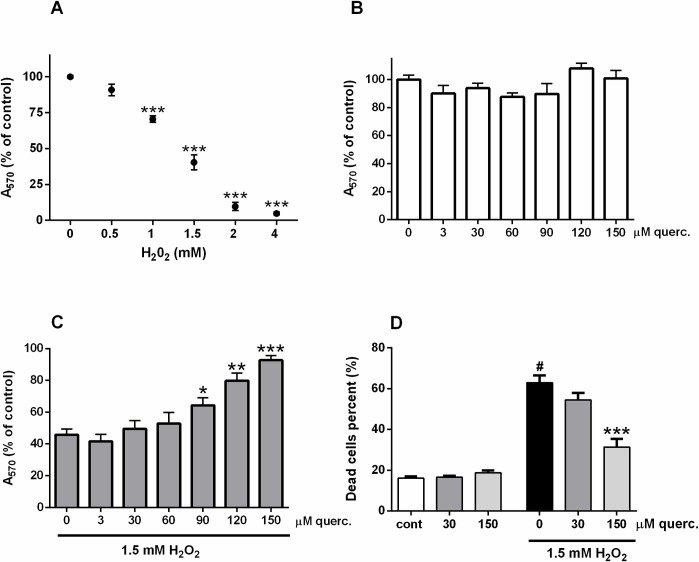
Quercetin did not affect an H_2_O_2_-induced decrease in content. At the end of the 24 h treatment, GSH content was depleted in P19 neurons exposed to 1.5 mM H_2_O_2_. Presence of quercetin did not modify the intracellular pool of GSH. Values represent the mean ± SEM of three independent experiments performed in triplicate. ^#^P < 0.0001 vs. vehicle-treated group (ONE-way ANOVA followed by Tukey's multiple comparison tests).

Exposure to H_2_O_2_ increases accumulation of ROS and induces oxidative stress. As we previously reported, quercetin prevents H_2_O_2_-provoked upregulation of ROS production [32]. Reduced glutathione (GSH), one of the major non-enzymatic intracellular antioxidants, also may modulate levels of ROS and participate in the oxidative stress response [33]. Here, we analysed levels of GSH following exposure to H_2_O_2_ and quercetin. As represented in Fig 2, we found depletion of GSH content in P19 neurons exposed to H_2_O_2_. Namely, detoxification of H_2_O_2_ by glutathione peroxidase utilizes two GSH molecules per reaction, consequently leading to GSH depletion. In addition, GSH decrease may result from GSH-mediated free radical scavenging, and through the formation of GSH-conjugates with various electrophilic compounds [33,34]. On the other hand, the neuroprotective effect of quercetin was not accompanied with the restoration of intracellular GSH content. Similar results were also demonstrated in mouse embryos exposed to H_2_O_2_ and quercetin [35]. We assumed that antioxidative activities of quercetin effectively substituted antioxidative functions of GSH and prevented the damaging effects of oxidative stress despite the reduced GSH content.

**Fig 3:**
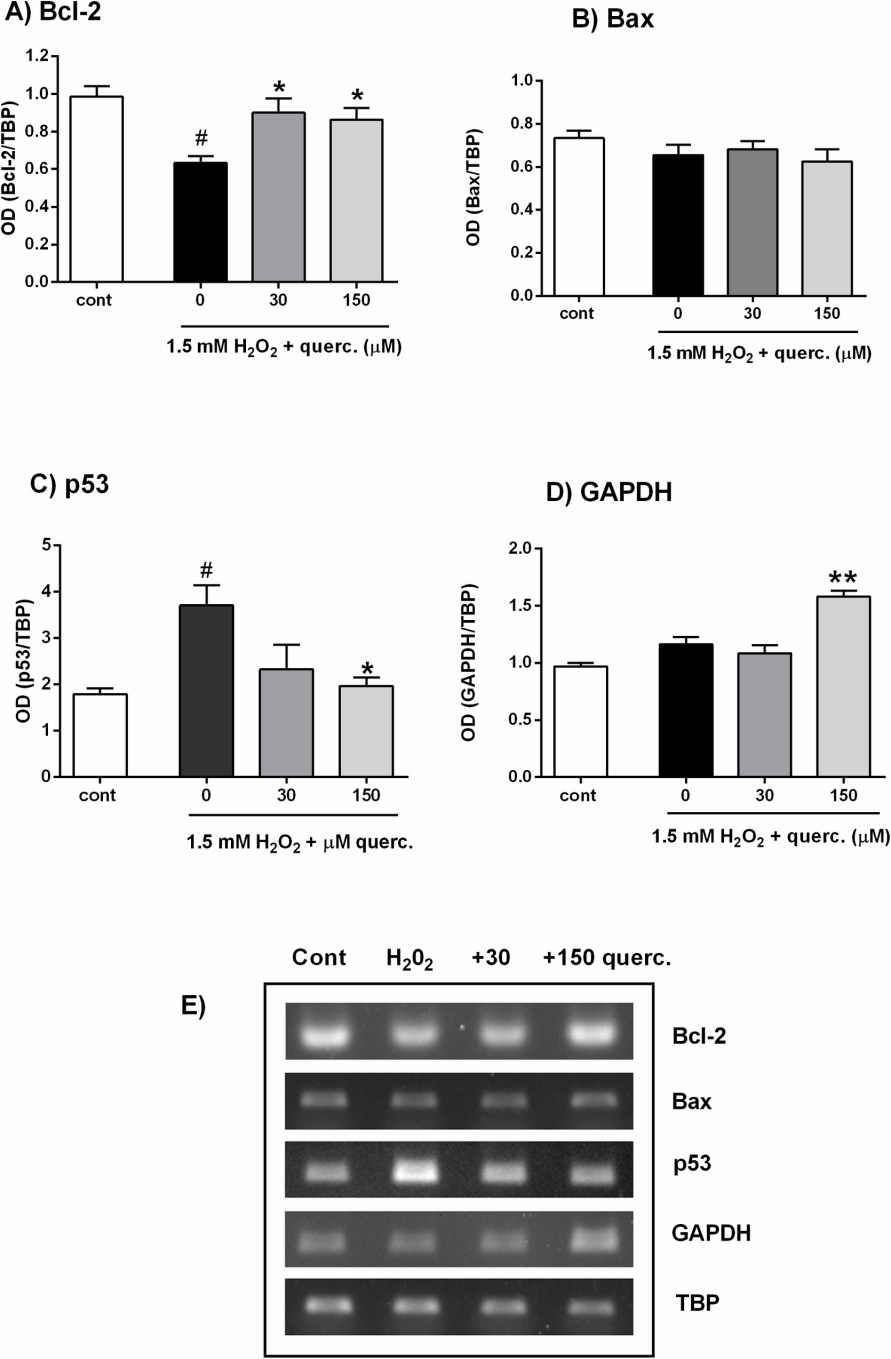
Effects of quercetin on Bcl-2, Bax, p53 and GAPDH mRNA expression during severe oxidative injury. P19 neurons were exposed to 1.5 mM H_2_O_2_ alone or in the presence of 30 and 150 μM quercetin. Total RNA was extracted and reverse transcribed into cDNA. The obtained cDNA was further amplified using specific primers. Following densitometric quantification, band intensities were normalized to the expression of housekeeping gene TBP. The data are expressed as means ± SEM from 3 independent RT-PCR analyses. ^#^P < 0.05 vs. cont; *P < 0.05, **P < 0.0001 vs. 0 group (ONE-way ANOVA and post-hoc Tukey’s multiple comparison test). Representative agarose gel electrophoresis is also shown.

P19 neurons exposed to high concentration of H_2_O_2_ die by caspase-independent apoptosis in combination with necrosis [32]. Neuronal death is commonly regulated by proteins from the Bcl-2 family. Bcl-2 and Bax are prototypical members of this family and their balance partly determines cell survival. As represented in Fig 3A, H_2_O_2_ reduced mRNA expression of antiapoptotic Bcl-2, whereas transcriptional expression of proapoptotic Bax was not affected (Fig 3B). Nevertheless, in the presence of quercetin transcriptional down-regulation of Bcl-2 was prevented (F(3,32) = 6.457, P = 0.0015), returning the overall Bcl-2/Bax ratio back to control values. Other studies also indicated that quercetin prevents neuronal death by increasing Bcl-2 levels [36,37]. We also found increased expression of transcription factor p53 during H_2_O_2_ treatment. Depending on the severity of the damage, p53 guides neuronal response to oxidative stress and determines the balance between survival and death in neurodegeneration [38]. Fig 3C indicates that H_2_O_2_-induced up-regulation of p53 expression was diminished in P19 neurons simultaneously exposed to quercetin (F(3,20) = 5.773, P = 0.0052). In neuroblastoma SK-N-MC cells, the neuroprotective effect of quercetin was also accompanied by the suppression of H_2_O_2_-induced p53 enhancement [9]. Similarly, in oxidative stress induced by transient focal cerebral ischemia and reperfusion, quercetin prevented neuronal loss by lowering p53 expression [39].

It has been shown that p53 may regulate caspase-independent cell death [40]. The main effector of caspase-independent death program is an apoptosis-inducing factor (AIF). Overexpression of Bcl-2 may prevent AIF release and consequent cell death [41]. Necrosis also may be prevented by Bcl-2 overexpression [42]. Hence, by preserving the p53 and Bcl-2 expression quercetin could contribute to the survival of P19 neurons at the level of apoptotic and necrotic death events.

We also analysed the expression of GAPDH mRNA. GAPDH is primarily viewed as a metabolic enzyme engaged in glycolysis, but it mediates numerous non-glycolytic functions, including the sensing of oxidative stress and induction of cell death [43,44]. Although exposure to H_2_O_2_ did not change GAPDH expression, the pro-survival effect of quercetin correlated with pronounced GAPDH up-regulation (Fig 3D). H_2_O_2_ readily stimulates conversion of cysteine sulfhydryl groups into disulfides and other oxidized species which may inhibit or promote disulfide bonding within or between redox-sensitive cytoplasmic proteins [45]. Different cysteine modifications were observed in the catalytic site of GAPDH, affecting its structure and function [44,46]. Thus, despite we did not find changes in GAPDH expression during oxidative stress, it is possible that P19 neurons have experienced loss of some GAPDH functions that were successfully restored by quercetin-induced up-regulation of GAPDH. This may include regulation of microtubules bundling and activation of p53-mediated death pathways [47]. Quercetin-induced changes in the expression profile of GAPDH were also demonstrated *in viv*o [48].

**Fig 4:**
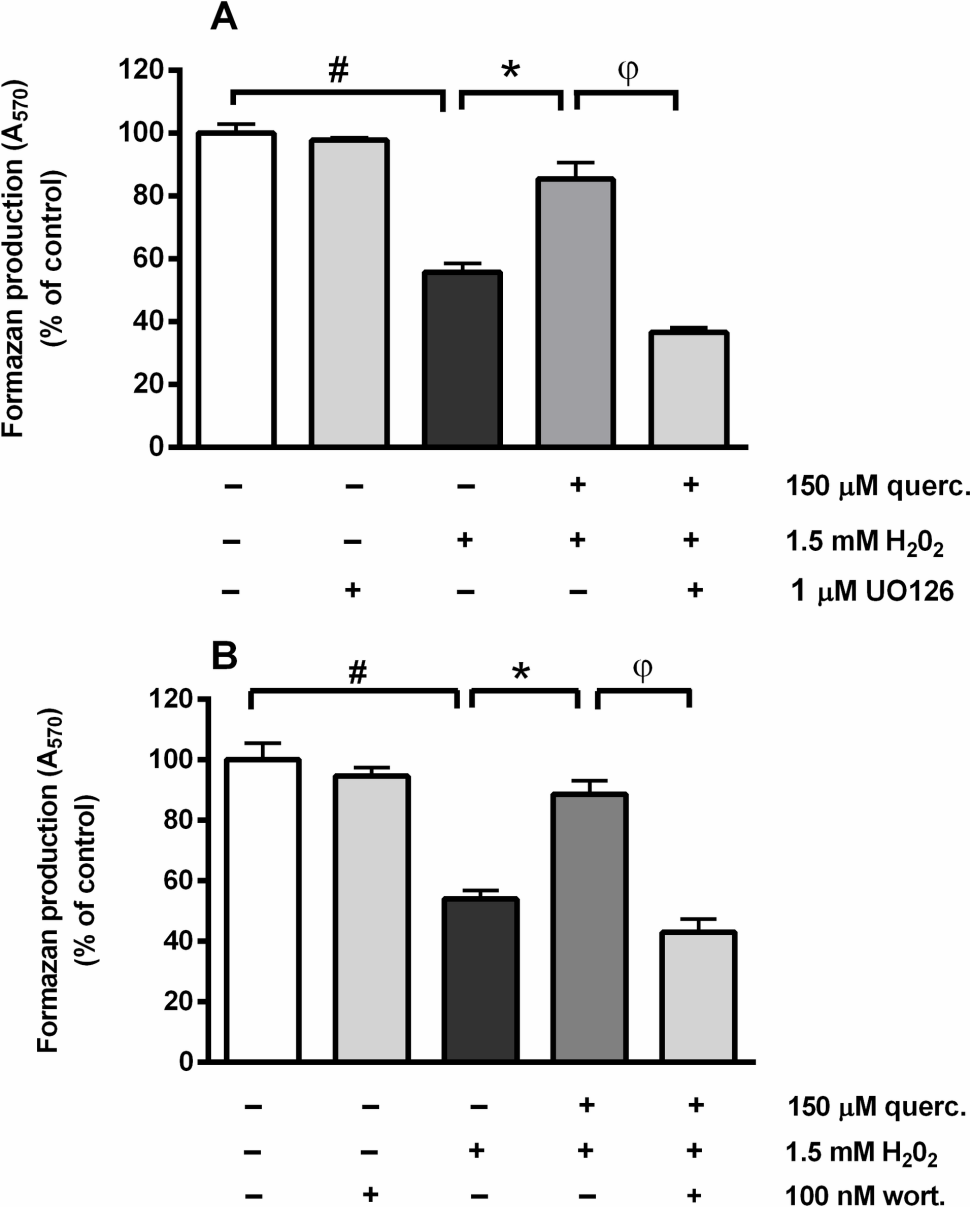
UO126 (inhibitor of the ERK1/2 pathway) and wortmannin (Akt/PKB inhibitor) prevented the neuroprotective effect of quercetin against oxidative injury. P19 neurons were treated with UO126 or wortmannin for 1 h prior to and during 24 h H_2_O_2_ treatment. UO126 applied at 1 μM concentration (upper graph), as well as 100 nM wortmannin (bottom graph) diminished survival of P19 neurons exposed simultaneously to 1.5 mM H_2_O_2_ and 150 μM quercetin for 24 h. Values are expressed as means ± SEM from three independent experiments performed in quadruplets. *P < 0.001 vs. 1.5 mM H_2_O_2_ alone; ^#^P < 0.0001 vs. cont; ^φ^P < 0.0001 vs. H_2_O_2_ + quercetin (one-way ANOVA followed by post hoc Tukey's test).

ROS-mediated activation of intracellular signalling pathways has an important role in determining neuronal survival [27]. On the other hand, flavonoids (including quercetin) may offer protection against oxidative damage by modulating intracellular cascades [12,49]. To investigate effects of quercetin on the modulation of intracellular signalling, P19 neurons were exposed to H_2_O_2_ and quercetin and two selected inhibitors: UO126 (an inhibitor of the Ras/Raf/MEK/ERK signalling pathway) or wortmannin (a covalent inhibitor of phosphoinositide 3-kinases (PI3K) that activates Akt/PKB). Both inhibitors were applied in a concentration that did not affect viability when applied alone. As represented in Fig 4, beneficial effects of quercetin on neuronal survival were abrogated in the presence of UO126 (F(4,10) = 83.20, P < 0.0001) and wortmannin (F(4,10) = 40.03, P < 0.0001). Thus, our results support the previous findings [10,18] which suggest that stimulation ERK1/2 and PI3K/Akt signalling underlies the protective effects of quercetin. In sympathetic neurons, it has been shown that activation of PI3K/Akt and MAPK/ERK pathways offers protection against p53-mediated cell death [50,51]. PKB/Akt acts upstream of p53 and suppresses transcriptional activation of p53-responsive genes [52] while signalling through both Akt and ERK1/2 pathways may trigger activity cyclic AMP regulatory-binding protein (CREB), thereby promoting transcription of antiapoptotic gene Bcl-2 [5,7].

### The new biophysical markers for identifying the neuroprotective effects of quercetin in H_2_O_2_-induced oxidative stress: the morphological and nanomechanical study

After we confirmed that quercetin promotes survival of P19 neurons in oxidative conditions, we used AFM to image neuronal morphology and to measure membrane roughness and nanomechanical properties, parameters that have potential in biomedical applications as specific biophysical markers for efficient screening of various cellular conditions, including oxidative stress. Our study was performed on P19 neurons fixed with 4% paraformaldehyde. It has been shown that fixation procedure may affect nanomechanical and structural properties of investigated cells [30]. However, as all neurons from control and treated groups have experienced the same fixation procedure, we assume that observed changes could be attributed to different drug treatments rather than fixation protocol. On the other hand, the imaging of living cells also may result in damage of the soft cell surface by the scanning tip, cell death, poor resolution and cell surface fluctuations [30,53]. Previous studies performed on live cortical neurons yielded slightly lower Young’s moduli in comparison with values obtained on fixed P19 neurons [54,55], but both types of neurons have similar elasticity maps [54,56]. Furthermore, by monitoring fragmentation of growth cones, similar properties have been found in living cells and cells fixed by different procedures (including 4% paraformaldehyde) [57]. Grimm et al. [58] studied mechanical ties of fixed cells and found that stiffness of fixed endothelial cells resembles the properties of the actin network of living cells. This indicates that fixed P19 neurons represent a good model system to study the effects of oxidative stress and the action of neuroprotective drugs at the nanoscale.

**Fig 5:**
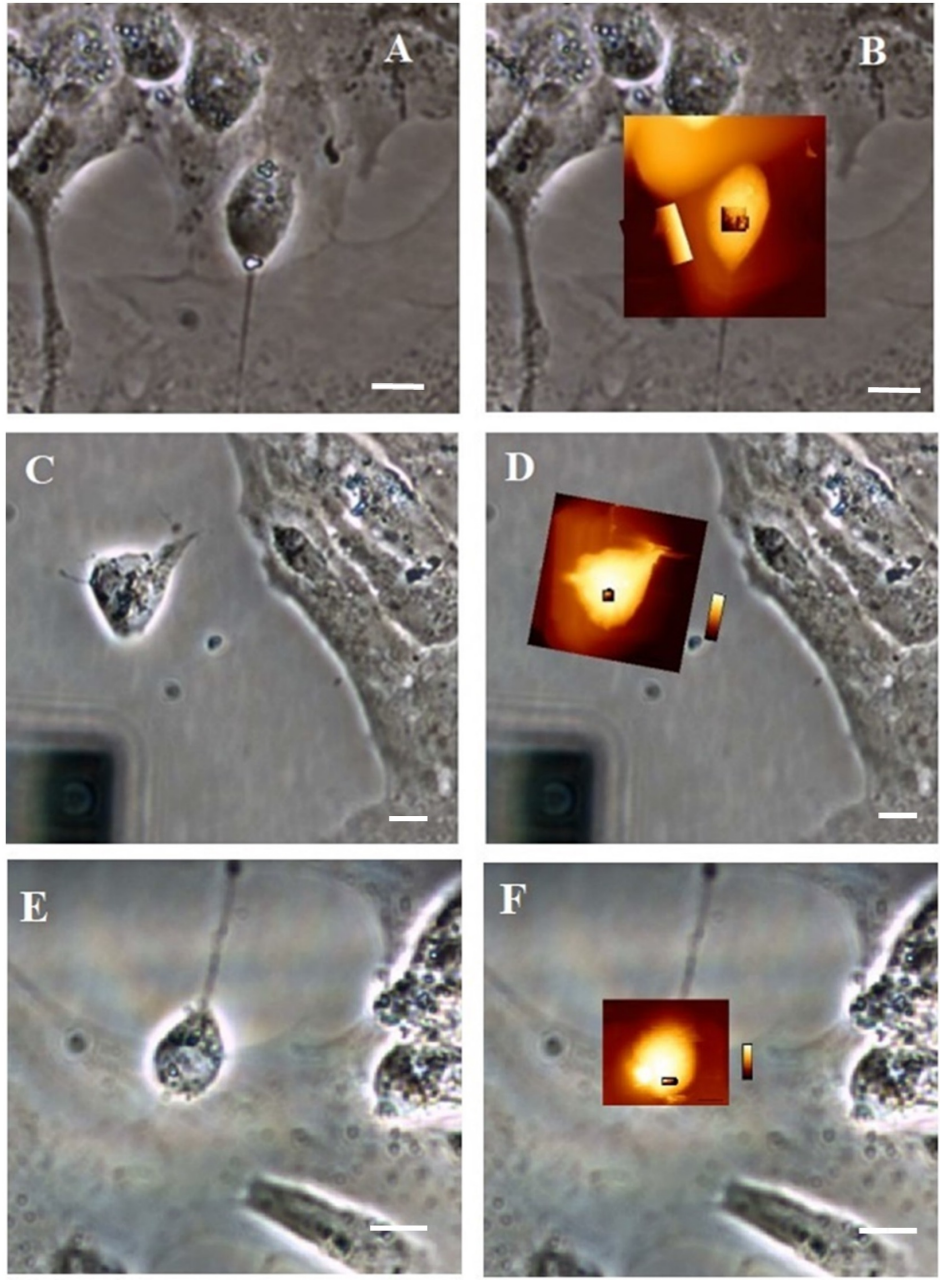
**Optical images (A, C, E) and overlay of optical and QI images (B, D, F) of control neuronal cells (A, B), H**_**2**_**O**_**2**_**-exposed neurons (C, D) and neurons simultaneously exposed to both quercetin and H**_**2**_**O**_**2**_ **(E, F).** Scale bar = 10 μm.

In order to more precisely identify morphological differences between control and treated groups, we introduced a multiple biophysical analysis by employing AFM. First, as represented in Fig 5, isolated control neurons displayed a slightly elongated soma shape (Fig 5A), whereas neurons exposed to H_2_O_2_ have irregular circular shape and degenerated cell bodies (Fig 5C). The morphological shape of P19 neurons simultaneously treated with quercetin and H_2_O_2_ was more regular, better resembling to control neurons (Figs 5A and 5E), indicating beneficial effects of quercetin on the preservation of neuronal morphology.

**Fig 6:**
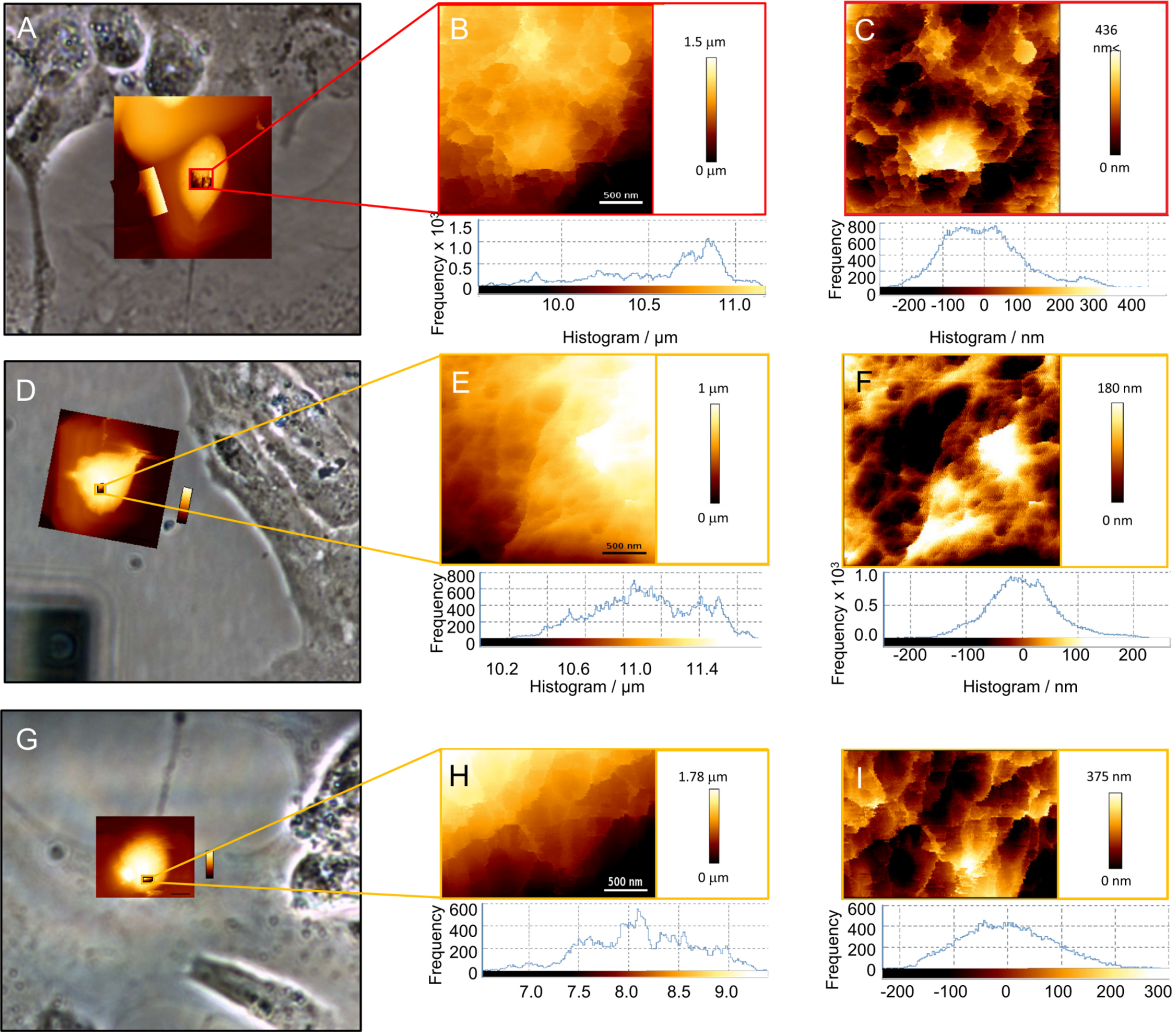
**Low-resolution inverted optical microscopic images on neuron soma control system (A), on neuron after the treatment with H**_**2**_**O**_**2**_ **(D) and after the simultaneous exposure to both quercetin and H**_**2**_**O**_**2**_ **(G).** The highest region of the soma was zoomed (B, E, H). The relative height differences between individual regions were consistent with data acquired from inverted optical microscopic images and indicated within histograms shown in (C, F, I). Scales are indicated. Frequency histograms of unfiltered normalized cut-off height (B, E, H bottom) and filtered normalized cut-off height (C, F, I bottom) for the line profile of a neuron.

Second, to better reveal effects of quercetin on structural changes at the nanoscale level, we measured Feret dimensions of neuronal somas and height of the different soma regions (Fig 6), considering these parameters as useful indicators of oxidative damage. Since most of the analysed neurons had somatic Feret dimensions between 10 μm and 25 μm, this size range was selected for all AFM studies. The values of neuronal soma Feret dimensions (FD_min_ and FD_max_) and height are presented in Table 2. The somatic height (h), as well as FD, particularly *FD*_*min*_ of H_2_O_2_-exposed neurons, were markedly lower in comparison to control. In addition, exposure to H_2_O_2_ provoked a marked decrease in the cell volume (Δ*V* = - 420 μm^3^), indicating significant rearrangement and conformational changes of cytoskeleton structure. During simultaneous treatment of neurons with H_2_O_2_ and quercetin, the observed cell volume change was approximately half of that obtained with H_2_O_2_, barely Δ*V* = - 205 μm^3^. Hence, with the applied approach using cell volume, we additionally demonstrated the protective effects of quercetin on neuronal morphology.

**Table 2:** Roughness, height and Feret dimension data from the histograms of the neuronal detail images below.

	*FD*_vmint_/ μm	*FD*_maxr_/ μm	*h*/ μm	*V*/ μm^3^	Average R_a_ / nm	R_q_ /nm	Average R_a_/ nm (quadratic plane fit)	R_q_/nm (quadratic plane fit)
Control	13.2±0.6	22.6±0.7	6.98±0.5	2080	280±6	351±7	77.5±0.9	99.1±0.4
*n* = 9								
H_2_O_2_	17.0±0.3	17.1±0.9	5.6±1.2	1660	206±3	267±5	49.0±0.5	64.1±0.1
*n* = 9								
Quercetin/ H_2_O_2_	17.5±0.6	16.5±0.4	6.5±0.2	1875	275±7	335±9	69.0±0.4	85.8±0.7
*n* = 9								

As evident in Fig 6B, distinct regions of control P19 neurons contain ruffling structures probably consisting of the assembly of diverse membrane proteins and membrane folding. Similar findings of fine ruffling formations have been observed in morphological studies of neuronal growth cone [22,59]. AFM imaging revealed that individual ruffling structures are of various sizes in control neurons (Fig 6E). Our values of ruffling heights are slightly lower than already observed heights of 160 nm for the P domain, 220 nm for the ruffling T zone, and 200 nm for the C domain [22]. Besides, the height histograms (S2 Fig) showed a distinct increase in the size of ruffling from 63.7% to 78.4%, in the range between 0 nm and 100 nm, while the proportion of higher protrusion with dimensions between 100 nm and 200 nm decreased from 29.7% to 21.6%. These membrane protrusions were significantly suppressed in H_2_O_2_-exposed neurons (Fig 6E) probably due to the formation of higher molecular weight components by cross-linking of membrane proteins [60]. The protective effect of quercetin was particularly evident in the height histogram and cross-section profile of neurons treated with both quercetin and H_2_O_2_ whose fine ruffling assemblies showed only minor modifications (for ruffling size between 0 nm and 100 nm 65.9%) in comparison with control neurons (Fig 6H, S2 Fig).

We also performed surface roughness analysis of the neuronal soma membrane to specifically evaluate changes in membrane surface topography. A membrane roughness is an important parameter in cell studies, also with great potential for medicinal applications. It indicates the deviation of the membrane surface topography from the ideally smooth surface. The roughness parameters described by *R*_a_ and *R*_q_ were derived from the raw height images and the height images treated with a quadratic plane fit. Nine neurons were analysed by randomly measuring several areas of each cell surface. The analysed regions of control cells were relatively small (2×2 μm^2^), but they appeared cone-shaped in cross-section profile, and their surfaces were relatively rough as shown in Table 2. The plane fit was used to subtract the curvature of the neuronal body which otherwise would influence the roughness data (Table 2). The plane fit more accurately revealed surface roughness. The scale-independent roughness parameters, obtained by computing the *R*_a_ value on a filtered profile, are comparable with the size of protein complexes and demonstrated membrane damage processes. While spherical crater edges in the control P19 neurons were very sharp and distinct (Fig 6B), in quercetin/H_2_O_2_ treated neurons they were pitted and became shallow (Fig 6H), and completely disappeared in H_2_O_2_-treated neurons (Fig 6E). In the presence of H_2_O_2_, it is obvious that decrease in roughness parameters indicates the discrete damage of cell membrane. By the addition of quercetin, changes of these parameters were markedly prevented, indicating only minor damage of the neuronal surface. Thus, reduced changes of roughness parameters indicate better structural preservation because of quercetin presence in the culturing medium. Hence, roughness data as a valuable biophysical marker showed a protective effect of quercetin against H_2_O_2_-induced oxidative injury at the membrane level.

Recently, Lee et al. [61] demonstrated that taxol-induced decrease in the roughness of neuroblastoma cells indicates an increase of membrane stiffness caused by microtubule translocation. In addition, they showed a time-lapse measurement of the decrease in membrane tension induced by a hypertonic solution that resulted in an increase in membrane roughness. On the contrary, our results showed that H_2_O_2_ induces a decrease in cell volume and therefore decrease in the membrane tension that resulted in the decrease of the roughness. Obviously, H_2_O_2_-induced oxidative stress in the highly organized cytoskeleton does not necessarily lead to the increase in membrane roughness. Therefore, membrane roughness can quantitatively describe cellular events at the nanoscale level including not only cytoskeletal alterations [62] and apoptotic processes [63] but also the organization of membrane components [64]. Due to the aforementioned formation of the higher molecular weight components by the crosslinking of membrane proteins, [60] the surface of H_2_O_2_-damaged membranes became markedly smoother (Fig 6F) in comparison to the rough surface of control neurons (Fig 6C). Membranes of neurons treated with both H_2_O_2_ and quercetin did not alter significantly, remaining their surface still rough (Fig 6H). Table 2 summarizes the roughness, height and Feret dimension values determined from the AFM height measurements on the highest domain of the neurons.

**Fig 7:**
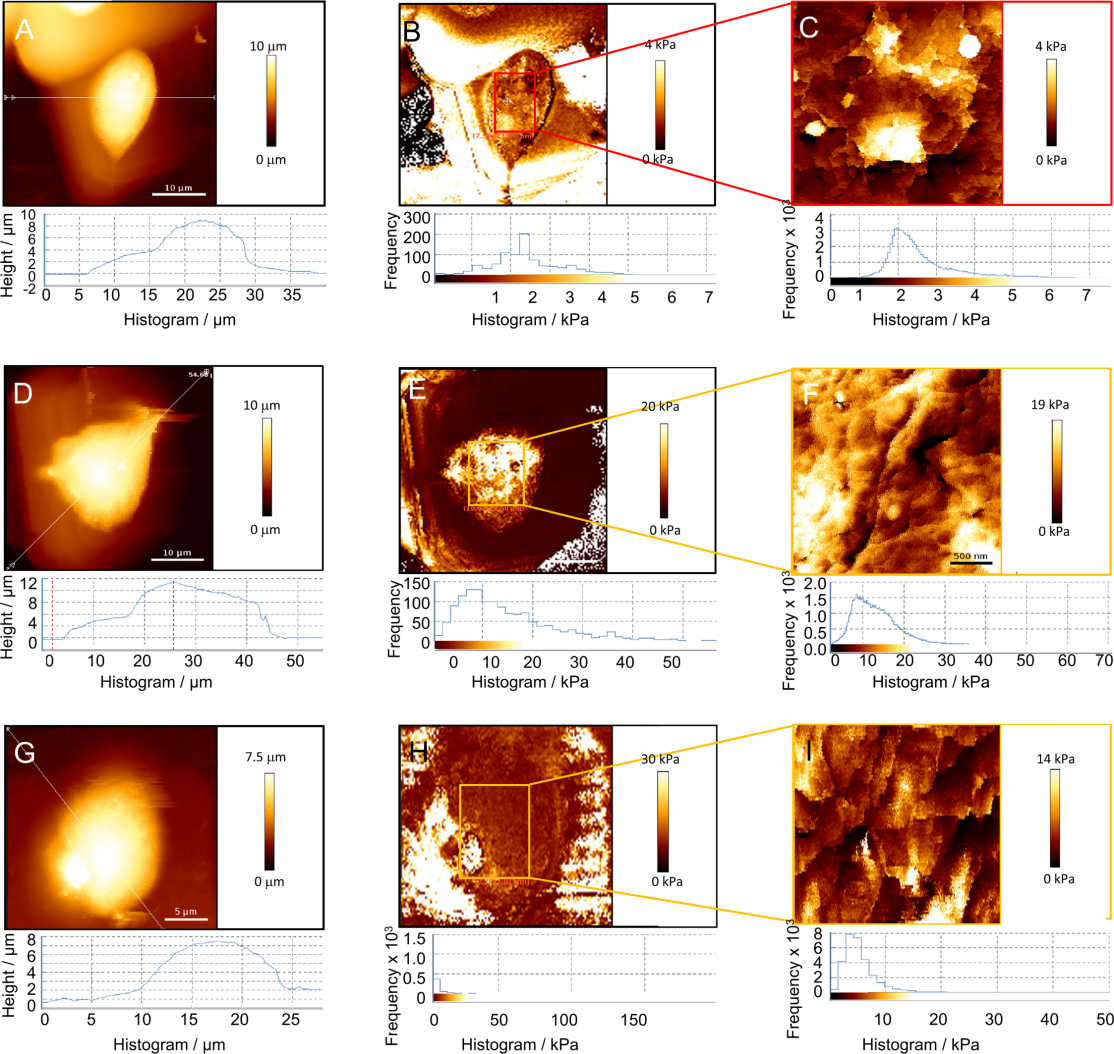
**The height topographic image of specific somatic regions in control system (A), H**_**2**_**O**_**2**_**-exposed neurons (D) and in neurons simultaneously exposed to both quercetin and H**_**2**_**O**_**2**_ **(G) determined by nanomechanical measurements using AFM.** The nanomechanical mapping and histogram of Young´ modulus of the specific somatic region in control system (B), H_2_O_2_-exposed neurons (E) and neurons simultaneously exposed to both quercetin and H_2_O_2_ (H). Colour-coded frame lines identified zoomed domain of control system (C) H_2_O_2_-exposed neurons (F) and neurons simultaneously exposed to both quercetin and H_2_O_2_ (I).

We further used AFM to analyse the local mechanical properties of specific somatic regions by performing nanomechanical measurements. AFM provides quantitative data of mechanical properties, as well as the direct relationship between mechanical and structural characteristics of neuronal cells [23]. Force-distance curves were acquired to determine the elastic moduli (Young’s moduli) of the different regions (Fig 7). Hertz’s model was used to fit the portions of the force curve and the resulting Young’s moduli are summarized in Table 3. As presented in the frequency histogram, significant variations in the elastic (Young’s) modulus were found between treated groups (Fig 7B, 7E and 7H, bottom). The Young’s modulus distribution of the control neuronal soma (within the colour-coded frame lines) varied between 0.5 kPa and 5 kPa with a maximum at 2.4±0.2 kPa. After H_2_O_2_ treatment distribution was spread to a much broader region from 0 kPa to 30 kPa and shifted its maximum towards higher value (14.7±0.5 kPa). The Young’s modulus distribution of the neurons concomitantly treated with quercetin and H_2_O_2_ varied at much narrow range (from 0 kPa to 15 kPa) and returned its maximum towards lower value (8.76±0.7 kPa). The same trend of membrane elasticity changes was observed from the detailed image of neurons, as shown in Table 3.

**Table 3:** Summarizing Young’s modulus data (average values from the histograms).

	Average value -overview image* /kPa	Average value -detail image* /kPa	*n*
Control	2.4±0.2	2.6±0.1	9
H_2_O_2_	14.7±0.5	13.0±0.3	9
Quercetin/ H_2_O_2_	8.7±0.7	8.1±0.2	9

*The Young’s modulus data were calculated using the Hertz fit for conical indenters using the same fit parameters for all samples.

In general, cell elasticity described by Young’s moduli could be considered as a measure to evaluate cell integrity and physiological state. In studied P19 neurons, changes in Young’s modulus values obtained from the different regions were not identical. If proteins are homogeneously distributed throughout the cytoplasm and the membrane, all regions of the neuronal soma possess the similar elasticity. However, the elasticity of P19 neurons, calculated from the overviewed and detailed images, were slightly different (Fig 7). While the Young modulus value (elasticity) in control neurons was homogeneously distributed (brown colour within a square in Fig 7B), the addition of H_2_O_2_ induced increase of stiffness that was reflected in the increase of the Young modulus value (white area within a square in Fig 7E). Besides, the obtained results indicate that the neuronal somas H_2_O_2_-treated cells contain heterogeneously arranged protein regions. These changes in Young’s moduli are probably determined not only with the local changes of the membrane structure but also with the local changes of the underlying cytoskeletal structure. The cross-linking of membrane proteins during oxidative stress might hinder the conformational change of membrane proteins and the conformational change of microfilaments within the cytoplasm [60]. Both might be the reason for the increased membrane Young´s modulus i.e. increased stiffness upon H_2_O_2_ treatment. However, in the presence of quercetin, only discrete increase in the neuron stiffness was observed (left side on the bottom square in Fig 7H), suggesting that Young modulus i.e. elasticity may serve as a biophysical marker for the estimation of the extension of the neuronal damage.

All these results suggest that quercetin offers protection towards certain mechano-chemical processes induced by an oxidative environment that affect cytoskeleton and membrane organization in the investigated regions of the neuronal somas.

The presented data obtained on fixed P19 neurons provide detailed evidence of morphological changes of cellular structures under different treatment regimes. First, we demonstrated inner structural reorganization of the cytoskeleton in oxidative conditions, manifested by increased cell somatic Feret dimensions, height and cell volume. The observed alterations in morphology were suppressed by quercetin presence confirming its neuroprotective role. Second, at the membrane level, the observed morphological changes were reflected in the distinct roughness values of treated neurons. The membrane roughness which originates from individual short and long protein ruffling structures [22] was decreased in P19 neurons exposed to H_2_O_2_ but turned back towards the initial state during simultaneous treatment with H_2_O_2_ and quercetin. H_2_O_2_ probably induces conformational changes of actin structures and promotes degradation of highly oriented bundles, consequently leading to their shortening which corresponds to the lower values of roughness parameters. Third, AFM was used to perform nanomechanical measurements in specific regions of P19 neurons. We observed that stiffer somatic regions simultaneously exhibited lower elasticity, i.e. higher Young’s modulus value. The stiffness (considered as the absence of the cell elasticity) of H_2_0_2_-treated neurons was markedly increased and then reduced almost to half in the presence of quercetin. Finally, we observed a strong correlation between cell stiffness and roughness parameters during H_2_O_2_-induced injury.

In conclusion, this AFM study represents the first detailed analysis of the protective effects of quercetin on neuronal membrane and cytoskeleton organization and in general, indicates a great potential of AFM in biomedical research. If combined with standard cellular and molecular methods, AFM can greatly upgrade monitoring of drug treatment outcomes on cellular physiology and morphology.

## Supporting information

S1 Fig **Uncropped original gels of RT-PCR products**: (A) Bcl-2, (B) Bax, (C) p53, (D) GAPDH, (E) TBP. For each gene (primer pair) we detected a single fluorescent band. The appropriate size of amplified PCR products was verified in experiments aimed to determine the log phase of PCR reactions. When setting the exposure time, we controlled the saturation to ensure that all bands were quantifiable. Photographs were exported as TIF image and cropped as indicated by red line
(TIF)

S2 Fig Histogram of the ruffling size in control, H_2_O_2_ and simultaneously treated with quercetin and H_2_O_2_ P19 neurons. (TIF)

S3 Fig Additional example images showing changes in morphology roughness and elasticity of cell membrane. Low-resolution inverted optical microscopic images on neuron soma control system (A), on neuron after the treatment with H_2_O_2_ (E) and after the simultaneous exposure to both quercetin and H_2_O_2_ (I). The cross section (B, F, J bottom) of height image (B, F, J). The highest region of the soma was zoomed (C, G, K). The relative height differences between individual regions were consistent with data acquired from inverted optical microscopic images and indicated within histograms shown in (D, H, L). Scales are indicated. Frequency histograms of unfiltered normalized cut-off height (C, G, K bottom) and filtered normalized cut-off height (D, H, L bottom) for the line profile of a neuron.
(TIF)

S4 Fig Additional example images showing changes in morphology roughness and elasticity of cell membrane. Low-resolution inverted optical microscopic images on neuron soma control system (A), on neuron after the treatment with H_2_O_2_ (E) and after the simultaneous exposure to both quercetin and H_2_O_2_ (I). The cross section (B, F, J bottom) of height image (B, F, J). The highest region of the soma was zoomed (C, G, K). The relative height differences between individual regions were consistent with data acquired from inverted optical microscopic images and indicated within histograms shown in (D, H, L). Scales are indicated. Frequency histograms of unfiltered normalized cut-off height (C, G, K bottom) and filtered normalized cut-off height (D, H, L bottom) for the line profile of a neuron.
(TIF)

S1 Table Additional raw data and statistical analysis of experimental measurements. (DOCX)
