# Correction: Atomic force microscopy reveals new biophysical markers for monitoring subcellular changes in oxidative injury: Neuroprotective effects of quercetin at the nanoscale

**DOI:** 10.1371/journal.pone.0212150

**Published:** 2019-02-06

**Authors:** Maja Jazvinšćak Jembrek, Josipa Vlainić, Vida Čadež, Suzana Šegota

Fig 2 is incorrect. Please see the correct Fig 2 here.

**Fig 2:**
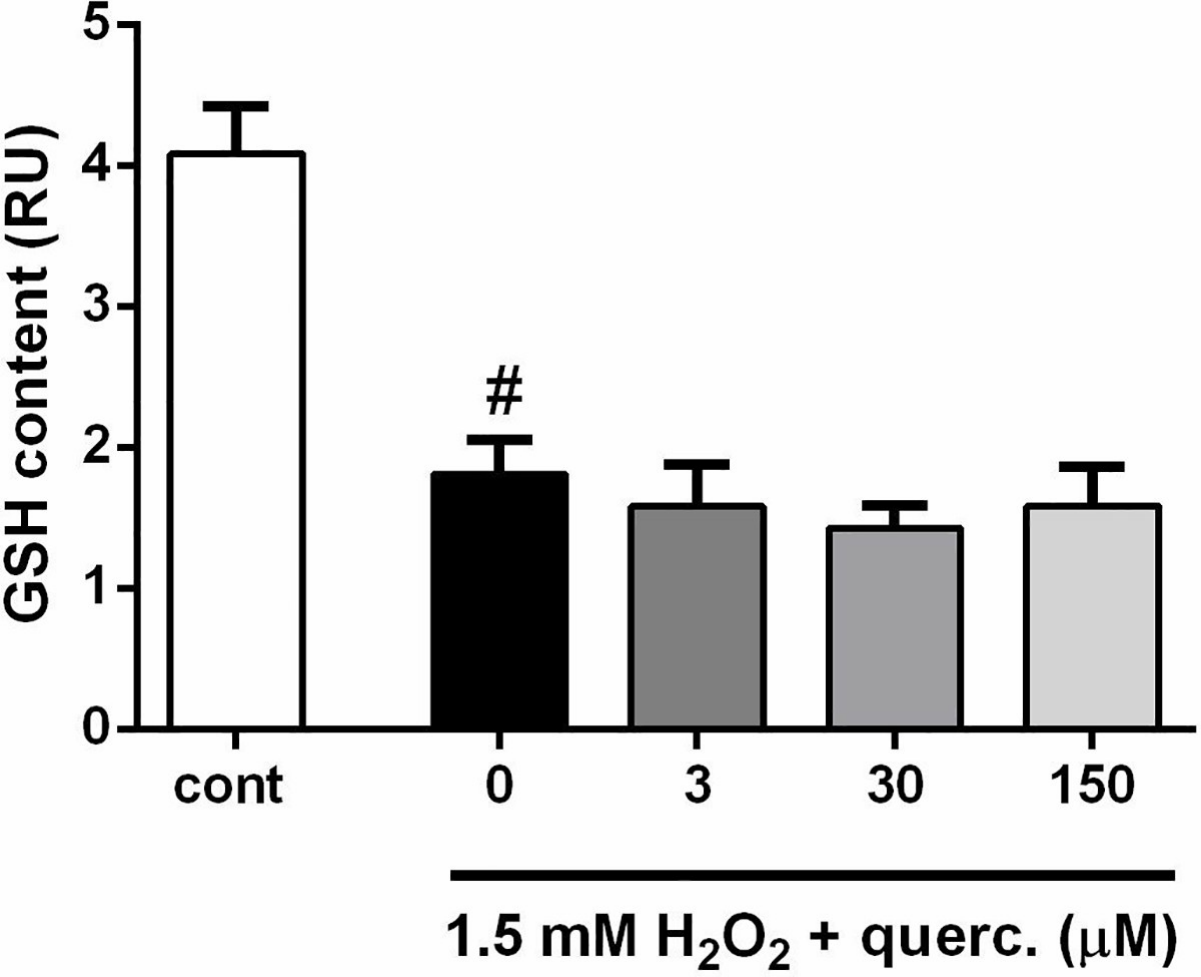
Quercetin did not affect an H_2_O_2_-induced decrease in content. At the end of the 24 h treatment, GSH content was depleted in P19 neurons exposed to 1.5 mM H_2_O_2_. Presence of quercetin did not modify the intracellular pool of GSH. Values represent the mean ± SEM of three independent experiments performed in triplicate. ^#^P < 0.0001 vs. vehicle-treated group (ONE-way ANOVA followed by Tukey's multiple comparison tests).

## References

[1] Jazvinšćak Jembrek M, Vlainić J, Čadež V, Šegota S (2018) Atomic force microscopy reveals new biophysical markers for monitoring subcellular changes in oxidative injury: Neuroprotective effects of quercetin at the nanoscale. PLoS ONE 13(10): e0200119 10.1371/journal.pone.0200119 30303965 PMC6179194

