# Supplementary figures and images for: Atomic force microscopy reveals new biophysical markers for monitoring subcellular changes in oxidative injury: Neuroprotective effects of quercetin at the nanoscale
Source: PLoS One. 2018 Oct 10;13(10):e0200119. doi: 10.1371/journal.pone.0200119 (PMC6179194; doi:10.1371/journal.pone.0200119)

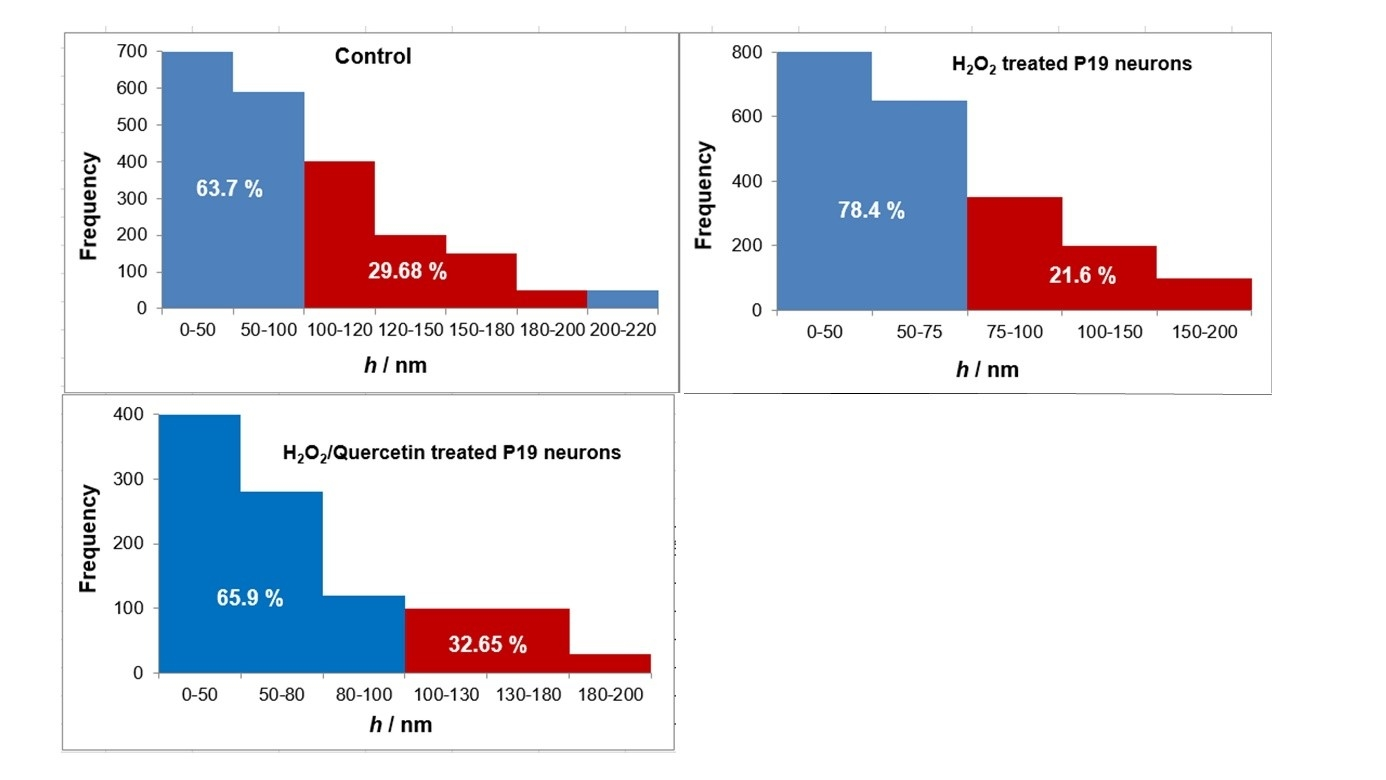

Supplement: S2 Fig — (TIF) [file pone.0200119.s002.tif]
