# Supplementary material for: Atomic force microscopy reveals new biophysical markers for monitoring subcellular changes in oxidative injury: Neuroprotective effects of quercetin at the nanoscale
Source: PLoS One. 2018 Oct 10;13(10):e0200119. doi: 10.1371/journal.pone.0200119 (PMC6179194; doi:10.1371/journal.pone.0200119)
Supplement: S1 Table — (DOCX) [file pone.0200119.s005.docx]

**S1 Table**. **Additional raw data and statistical analysis of experimental measurements.**

| **Control** | | | | | | |  |
| --- | --- | --- | --- | --- | --- | --- | --- |
| **Feret *d* _min_/μm** | **Feret d _max_/μm** | **h/μm** | **R_a_/nm** | **Rq/nm** | **R_a_ QPF nm** | **R_q_ QPF/ nm** |  |
| 13,3 | 22,6 | 6,2 | 286 | 340 | 77 | 99,2 |  |
| 13,5 | 22,7 | 7,5 | 276 | 356 | 77 | 99,4 |  |
| 12,9 | 21,9 | 7,3 | 269 | 348 | 77 | 98,1 |  |
| 12,9 | 22,9 | 6,4 | 282 | 352 | 77,5 | 99,5 |  |
| 13,8 | 23,1 | 7,1 | 285 | 358 | 78 | 99,2 |  |
| 13,9 | 22,1 | 6,8 | 282 | 360 | 78 | 98,7 |  |
| 12,7 | 21,8 | 6,6 | 283 | 346 | 79 | 98,8 |  |
| 11,9 | 23,3 | 7,3 | 278 | 340 | 76 | 99,4 |  |
| 13,7 | 23,2 | 7,6 | 286 | 355 | 78 | 99,2 |  |
| **13,17778** | **22,62222** | **6,977778** | **280,7778** | **350,5556** | **77,5** | **99,05556** | **Average** |
| **0,643774** | **0,567401** | **0,47088** | **5,585199** | **7,468452** | **0,8660254** | **0,447524** | **SD** |
|  | | | | | | | |
| **H_2_O_2_** | | | | | | |  |
| **Feret *d* _min_/μm** | **Feret *d* _max_/μm** | **h/μm** | **R_a_/nm** | **R_q_/nm** | **R_a_ QPF /nm** | **R_q_ QPF/ nm** |  |
| 17,3 | 17,35 | 5,2 | 208 | 268 | 48,4 | 64 |  |
| 17,3 | 19,4 | 5,5 | 208 | 272 | 49,2 | 64,05 |  |
| 17 | 17,1 | 7,3 | 207 | 274 | 49,5 | 64,2 |  |
| 17,1 | 17,15 | 6,7 | 203 | 264 | 48,5 | 64,2 |  |
| 16,7 | 16,75 | 7,4 | 204 | 262 | 48,3 | 64,1 |  |
| 16,2 | 16,25 | 3,9 | 206 | 263 | 49,2 | 64 |  |
| 16,9 | 16,95 | 3,7 | 209 | 259 | 49,5 | 64 |  |
| 17 | 17,05 | 6,3 | 200 | 265 | 49,2 | 64,2 |  |
| 17,1 | 17,15 | 4,8 | 205 | 272 | 48,9 | 64,1 |  |
| **16,95556** | **17,23889** | **5,644444** | **205,5556** | **266,5556** | **48,966667** | **64,09444** | **Average** |
| **0,339526** | **0,869906** | **1,374874** | **2,877113** | **5,198825** | **0,4636809** | **0,088192** | **SD** |
|  | | | | | | | |
| **Quercetin/ H_2_O_2_** | | | | | | |  |
| **Feret *d* _min_/μm** | **Feret *d* _max_/μm** | **h/μm** | **R_a_/nm** | **R_q_/nm** | **R_a_ QPF /nm** | **Rq QPF/ nm** |  |
| 16,1 | 16,6 | 6,3 | 275 | 331 | 69 | 85 |  |
| 16,3 | 16,9 | 6,48 | 286 | 349 | 69,1 | 86,2 |  |
| 17 | 18,2 | 6,5 | 285 | 350 | 69,2 | 85,7 |  |
| 16,5 | 17,6 | 6,51 | 270 | 330 | 68,8 | 86,5 |  |
| 17,1 | 18 | 6,5 | 265 | 327 | 68,5 | 86,7 |  |
| 17 | 18,1 | 6,7 | 269 | 328 | 68,4 | 86,2 |  |
| 16,1 | 17 | 6,39 | 276 | 335 | 69,3 | 85,1 |  |
| 16,3 | 17,4 | 6,8 | 269 | 328 | 68,2 | 85 |  |
| 16,5 | 17,4 | 6,47 | 277 | 335 | 69,3 | 86 |  |
| **16,54444** | **17,46667** | **6,516667** | **274,6667** | **334,7778** | **68,866667** | **85,82222** | **Average** |
| **0,371517** | **0,563471** | **0,150416** | **7,26292** | **8,828615** | **0,4123106** | **0,65532** | **SD** |
